# Validation of the reading the mind in the eyes test requires an interpretable factor model

**DOI:** 10.1073/pnas.2303706120

**Published:** 2023-12-18

**Authors:** Wendy C. Higgins, Victoria Savalei, Vince Polito, Robert M. Ross

**Affiliations:** ^a^School of Psychological Sciences, Macquarie University, North Ryde, NSW 2109, Australia; ^b^Department of Psychology, University of British Colombia, Vancouver, BC V6T 1Z4, Canada; ^c^Department of Philosophy, Macquarie University, North Ryde, NSW 2109, Australia

In a recent study of the Reading the Mind in the Eyes Test (Eyes Test), Greenberg et al. (1) claim to have provided “robust validation” for the test’s psychometric properties, supported by “acceptable-to-good reliability of the Eyes Test across datasets and countries” (p. 8). However, we have two critical concerns.

First, Greenberg et al. (p. 3) state that the reliability of the Eyes Test was “acceptable-to-good” based on both coefficient omega total (ω_t_; range: 0.74 to 0.80) and coefficient omega hierarchical (ω_h_; range: 0.32 to 0.50). However, this is incorrect. The ω_h_ values are substantially lower than acceptable (i.e., ω_h_ ≪ 0.80; 2, 3). Both ω_h_ and ω_t_ are based on bifactor modelling. Bifactor models comprise a general factor that influences all items (which should capture the target construct) and several group factors, which are independent of the general factor and typically represent construct-irrelevant variance. While ω_h_ captures the proportion of variance in the test scores due to the general factor only (i.e., variance related to the target construct), ω_t_ is based on variance due to both the general factor and the (usually construct-irrelevant) group factors. Critically, when these two estimates differ substantially, ω_h_ better represents the extent to which the sum scores reflect the target construct (4–6). In addition, only ω_t_ and not ω_h_ values were reported for individual countries, making it impossible to identify which, if any, countries had acceptable ω_h_ values.

Second, Greenberg et al. provide no details of the bifactor model used to calculate ω_t_ and ω_h_. Details of the fitted model, including the estimated solution, need to be reported because acceptable omega estimates can hide poor solutions, such as uninterpretable loading patterns, common in these models (6). However, even basic properties of the model, such as the number and composition of group factors (and whether they were the same across countries), were not reported. Critically, given the low values of ω_h_ relative to ω_t_, group factors may have contributed substantially to the reported effects. Details of the bifactor model are necessary to evaluate the extent to which reported effects (e.g., sex differences) are due to the target construct of interest (theory of mind) rather than group factors. Further, when comparing performance across samples (e.g., by sex), the same bifactor model should be fit to each sample to establish that the number of group factors and the indicators they comprise are the same (i.e., configural invariance) (6). Higher levels of measurement invariance (e.g., metric and scalar) would need to be established to claim that differences in performance are due to the target construct rather than, for example, differing strengths of group factors across populations (7).

Given serious concerns about the Eyes Test as a measure of theory of mind expressed here and elsewhere (8–10), we strongly caution against interpreting Greenberg et al.’s study as providing “robust validation for the psychometric properties of the Eyes Test” (p. 8) or evidence for sex differences in theory of mind ability.
